# Comparison of causal forest and regression-based approaches to evaluate treatment effect heterogeneity: an application for type 2 diabetes precision medicine

**DOI:** 10.1186/s12911-023-02207-2

**Published:** 2023-06-16

**Authors:** Ashwini Venkatasubramaniam, Bilal A. Mateen, Beverley M. Shields, Andrew T. Hattersley, Angus G. Jones, Sebastian J. Vollmer, John M. Dennis

**Affiliations:** 1 The Alan Turing Institute, British Library, 96 Euston Road, London, NW1 2DB UK; 2 University College London, Institute of Health Informatics, 222 Euston Rd, London, NW1 2DA UK; 3 University of Exeter Medical School, Institute of Biomedical & Clinical Science, RILD Building, Royal Devon & Exeter Hospital, Barrack Road, Exeter, EX2 5DW UK; 4 Department of Statistics, University of Warwick, Coventry, CV4 7AL UK

**Keywords:** Precision medicine, Treatment effect heterogeneity, Heterogeneous treatment effects, Counterfactual prediction, Machine learning, Causal forest, Type 2 diabetes, Generalized random forests, Treatment selection

## Abstract

**Objective:**

Precision medicine requires reliable identification of variation in patient-level outcomes with different available treatments, often termed treatment effect heterogeneity. We aimed to evaluate the comparative utility of individualized treatment selection strategies based on predicted individual-level treatment effects from a causal forest machine learning algorithm and a penalized regression model.

**Methods:**

Cohort study characterizing individual-level glucose-lowering response (6 month reduction in HbA1c) in people with type 2 diabetes initiating SGLT2-inhibitor or DPP4-inhibitor therapy. Model development set comprised 1,428 participants in the CANTATA-D and CANTATA-D2 randomised clinical trials of SGLT2-inhibitors versus DPP4-inhibitors. For external validation, calibration of observed versus predicted differences in HbA1c in patient strata defined by size of predicted HbA1c benefit was evaluated in 18,741 patients in UK primary care (Clinical Practice Research Datalink).

**Results:**

Heterogeneity in treatment effects was detected in clinical trial participants with both approaches (proportion predicted to have a benefit on SGLT2-inhibitor therapy over DPP4-inhibitor therapy: causal forest: 98.6%; penalized regression: 81.7%). In validation, calibration was good with penalized regression but sub-optimal with causal forest. A strata with an HbA1c benefit > 10 mmol/mol with SGLT2-inhibitors (3.7% of patients, observed benefit 11.0 mmol/mol [95%CI 8.0–14.0]) was identified using penalized regression but not causal forest, and a much larger strata with an HbA1c benefit 5–10 mmol with SGLT2-inhibitors was identified with penalized regression (regression: 20.9% of patients, observed benefit 7.8 mmol/mol (95%CI 6.7–8.9); causal forest 11.6%, observed benefit 8.7 mmol/mol (95%CI 7.4–10.1).

**Conclusions:**

Consistent with recent results for outcome prediction with clinical data, when evaluating treatment effect heterogeneity researchers should not rely on causal forest or other similar machine learning algorithms alone, and must compare outputs with standard regression, which in this evaluation was superior.

**Supplementary Information:**

The online version contains supplementary material available at 10.1186/s12911-023-02207-2.

## Background

Randomized controlled trials (RCTs) are the gold standard for understanding the effect of treatments on clinical outcomes. Average treatment effects from RCTs are then used to support evidence-based clinical decision making for individual patients. This application of a population-level result to individual treatment selection may result in sub-optimal decision making, as the average treatment effects may only represent the individual experience of a subset of patients [1]. As a result, there is great interest in developing precision medicine approaches to treatment, by characterizing patient sub-populations for which a treatment is most beneficial, or harmful. Such variability in patient level outcomes is known as treatment effect heterogeneity [2, 3], and is often obscured by average treatment effects. Importantly, if differences are clinically significant, characterizing treatment effect heterogeneity may allow specific treatments to be targeted at patients most likely to benefit.

Methods to evaluate treatment effect heterogeneity are not well established. One-variable-at-a-time subgroup analysis approaches have been shown to be rarely replicable due to low power, and will miss treatment effect heterogeneity induced by complex covariate relationships [3]. Traditional regression-based models can be used to estimate treatment effect heterogeneity across multiple variables by defining potential treatment-covariate interactions for each covariate of interest, but require these covariates to be specified by the analyst. Results may in particular be subject to the risk of Type I Error rate inflation (false positives) with small sample sizes, which may not be solved by penalized or shrinkage methods [4]. Recently, machine learning algorithms, in particular causal forest, have been developed to specifically assess treatment effect heterogeneity and represent a data-driven alternative to regression-based approaches potentially overcoming challenges associated with reliance on manual input and prespecification of treatment-covariate interaction terms [5, 6]. Whilst in a recent simulation study causal forest outperformed a two-step regression approach that estimated differential treatment effects by building separate models in each treatment arm [7], its comparative utility relative to regression for the purpose of treatment selection informed by treatment effect heterogeneity has not, to our knowledge, been previously assessed in applied studies using clinical data [8].

This issue of sub-optimal (personalized) decision making is potentially evident in the pharmacological management of Type 2 diabetes; a heterogenous chronic condition with multiple treatment options prescribed with the primary clinical purpose of lowering blood glucose (glycated hemoglobin [HbA1c]) levels. SGLT2-inhibitors (SGLT2-i) and DPP4-inhibitors (DPP4-i) are two commonly prescribed glucose-lowering treatment options [9], recommended after metformin in type 2 diabetes clinical guidelines [10]. Whilst RCT data suggest that the glucose-lowering efficacy of both treatments is on average similar [11], treatment effect heterogeneity is plausible due to the marked variation in the clinical characteristics of people with type 2 diabetes, and the differing mechanisms of action of the two drug classes [12]. As such, our primary objective in this study was to compare individualized treatment selection strategies based on predicted treatment effects from a causal forest algorithm and a penalized regression model, using the clinically relevant context of selecting between SGLT2-i and DPP4-i therapy for people with type 2 diabetes.

## Methods

### Overview

Two treatment effect heterogeneity models (causal forest and penalized regression) were developed to predict HbA1c-lowering efficacy with SGLT2-i and DPP4-i therapy using individual-level participant data from two large RCTs. Performance of individualized treatment selection strategies derived from each model was evaluated in routine clinical data.

### Data sources and Handling

#### Clinical trial data (development dataset)

Individual participant data from 2 active comparator glucose-lowering efficacy RCTs of SGLT2-i (Canagliflozin) and DPP4-i (Sitagliptin) therapy (2010–2012) in people with type 2 diabetes were accessed from the Yale University Open Data Access Project (https://yoda.yale.edu/). Data on participants randomized to either SGLT2-i or DPP4-i in the CANTATA-D and CANTATA-D2 were pooled for analysis; these trials differed only in background glucose-lowering therapy not in any other inclusion criteria. Trial results to compare the average HbA1c-lowering efficacy of the two therapies have been previously published [13, 14].

#### Routine clinical data (test dataset)

Anonymized primary care electronic health records were extracted from UK Clinical Practice Research Datalink (CPRD) GOLD [15]. New users of SGLT2-i and DPP4-i therapies (i.e. patients initiating one of these therapies for the first time) after January 1st, 2013, were identified, following our previously published protocol [16]. We then excluded patients prescribed a SGLT2-i or DPP4-i as first-line treatment (as this is outside of treatment guidelines) [10], patients co-treated with insulin as response will reflect (poorly recorded) insulin titration and participants in the RCTs were all non-insulin treated, patients with eGFR < 45 (where SGLT2-i prescription was usually contraindicated at the time of data collection), patients with a missing baseline HbA1c (the major determinant of HbA1c response) or a baseline HbA1c < 53 (as this is threshold for glucose-lowering medication initiation in clinical guidelines) or presenting with severe hyperglycemia of ≥ 120 mmol/mol. Baseline HbA1c was defined as the closest HbA1c to drug initiation within –91/ + 7 days.

#### Predictors

Across both data sources, the following clinical features were extracted for each individual: baseline HbA1c, age at treatment, sex, estimated glomerular filtration rate (eGFR), Alanine Aminotransferase (ALT), body mass index (BMI), High-density lipoprotein cholesterol (HDL-c), Low-density lipoprotein cholesterol (LDL-c), Triglycerides, Albumin, and Bilirubin. These features were selected due to their availability in a majority of individuals in both the trial and routine data. Diabetes duration was redacted from the RCT data so was not evaluated. Ethnicity was not evaluated as a candidate predictor as trial participants were predominantly of White ethnicity. In CPRD, where a systematic baseline assessment of clinical features was not available, for all biomarkers we used the most recent value in the 2 years prior to drug initiation available in the primary care record.

#### Confounders

In CPRD, we also identified potential confounders, comprising the number of current, and ever, prescribed glucose-lowering drug classes, ethnicity (white, Asian, black, mixed or other), smoking status (active, ex, or non smoker), diabetes duration, history of hypertension, cardiovascular disease, heart failure, chronic kidney disease, or microvascular complications (neuropathy, nephropathy, or retinopathy), and history of non-adherence on first line therapy, with non-adherence defined as previously published [17].

#### Missing data handling

In the trials, missing values in all covariates were imputed using missForest, a random forest based imputation method [18]. For validation of the model developed in the trials in CPRD, we conducted complete case analysis, as missing values were considered likely to be missing not at random [19].

### Statistical modelling

Two treatment effect heterogeneity models were developed using RCT training data. During model development the prediction target was the achieved HbA1c 6 months after drug initiation (a measure of glucose-lowering efficacy), evaluated as a continuous measure. In the trials, this was defined as the last-observation-carried-forward HbA1c from 3-months if the 6-month value was not available. In CPRD, this was defined as the closest HbA1c to 6 months (within 3–15 months) after initiation, on unchanged glucose-lowering therapy. Subsequently, utility of the models for selecting optimal treatment for patients was evaluated in routine clinical electronic medical record data using a novel framework [12].

#### Model development in trial data: penalized regression

A multivariable linear regression model was fitted to the training dataset composed of all baseline features (see Predictors section), the outcome and the treatment indicator. Each of the eleven continuous baseline features was modelled as a 3-knot restricted cubic spline to allow for non-linearity. Interaction terms for each baseline feature:treatment indicator pair were included to estimate treatment effect heterogeneity. No variable selection was applied, but optimal penalty factors, based on Akaike information criterion (AIC), were estimated separately for main effects, non-linear effects, and interaction terms, using an approach similar to ridge regression (*pentrace* function in R package *RMS*) [20]. We compared performance of this model with an alternative of Lasso regression, fitted using the same baseline feature set*.* Optimism-adjusted model fit (R^2^), root mean square error (RMSE), the calibration slope, and calibration-in-the-large were estimated, although these test the ability of a model to predict the outcome, and are therefore of limited use when evaluating treatment effect heterogeneity. Relative feature importance, in terms of treatment effect heterogeneity, was assessed by ranking features by the proportion of chi-squared explained by the interaction term for that feature, with bootstrapped confidence intervals.

#### Model development in trial data: causal forest

A causal forest model was also fitted over the training dataset. The causal forest model was built over 5000 causal trees and used default tuning parameters for growing the many tree structures. Tuning parameters used for growing an individual causal tree included setting a minimum of ten patients within a determined subgroup and splitting the training dataset equally into two separate random samples for first determining the tree structure, and then utilising the second sample for treatment effect estimation at each determined subgroup. To assess the sensitivity of the model to the number of casual trees we repeated the model building step with 50, 100, 200, 500, 1000, 2500, 7500, and 10,000 trees. Variable importance measures computed from trees in the forest highlight the covariates selected most frequently by the model. However, classification and regression trees (CART) and associated ensemble structures (e.g., random forests) have been shown to be biased towards splitting over covariates that offer many potential values to split on (e.g., continuous covariates) as compared to covariates with few categories (e.g., binary covariates). To account for this problem of biased variable selection, adjusted feature importance in the form of *p*-values were determined using a permutation-based test [21, 22]. A *p*-value for each covariate is computed by determining the proportion for which importance measures from forest models over permuted responses are greater than the measure obtained for a forest using an unpermuted response.

#### Model evaluation in routine clinical data

Utility of the two treatment effect heterogeneity modelling approaches for selecting the likely most effective therapy for patients was tested in CPRD. The first step was to estimate the difference in the in predicted HbA1c outcome (the conditional average treatment effect; Table 1) for each patient using both models. The accuracy of the CATE cannot be evaluated at the patient-level (as patients receive either SGLT2-i or DPP4-i but not the other). However, it can be used to define and test a treatment selection decision rule in patient strata defined by the difference in predicted HbA1c outcome, as follows: For each model, the difference in HbA1c outcome was estimated for each patient. For penalized regression this was the difference in predicted HbA1c outcome on the two therapies. In the causal forest algorithm, the difference in HbA1c outcome is explicitly estimated. Strata were then defined by decile of predicted difference in predicted HbA1c outcome, and by clinically defined HbA1c cut-offs of predicted difference in HbA1c outcome (SGLT2i benefit: ≥ 10, 5–10, 3–5, 0–3 mmol/mol; DPP4i benefit: ≥ 5, 3–5, 0–3 mmol/mol), with 3 mmol/mol used widely as minimally important difference in clinical trials [23]. To compare performance of each model, we tested whether within-strata HbA1c outcome differences were consistent with predictions. Linear regression models were used to contrast HbA1c outcome in concordant (i.e. therapy received is the therapy predicted to have greatest HbA1c lowering) versus discordant (i.e. therapy received is the predicted non-optimal therapy) subgroups. As CPRD patients were not randomized to treatment, models were adjusted for all features used in the treatment selection model (see Predictors section), and confounding factors (see Confounders section). Statistical analysis used R software, with causal forest fitted using the *grf* package [24].

**Table 1:** Primer on Conditional Average Treatment Effect (CATE) estimation

Evaluation framework
In a potential outcomes framework, the causal effect of a treatment on a patient is defined by the difference in outcomes, where the outcomes are obtained for two different treatment assignments. The conditional average treatment effect (CATE) is defined as the average over individual treatment effects for a subpopulation determined by specific patient characteristics. The estimation of such subgroup-specific treatment effects has traditionally relied on a manual comparison of pre-defined patient sub-populations. However, this is not necessarily possible for subgroups determined by unknown covariate relationships or for higher-dimensional datasets. We evaluate two different methods that are able to estimate conditional average treatment effects, which represent differential patient responses to a treatment allocation
Penalized regression
Standard maximum likelihood regression models can estimate CATE by including treatment-by-covariate interaction terms. For each covariate, the interaction term coefficient(s) represent the estimated differential treatment effect associated with that covariate. The model can then be used to predict the counterfactual outcome on each therapy, conditional on the features included as interaction terms. The difference between the predicted outcome on each therapy provides an estimate of the patient-level treatment effect. Penalized regression can be used to reduce overfitting and potentially improve prediction in new data
Causal forest
Causal forest is a data-driven ensemble method built over many individual causal trees to estimate the CATE [6]. A causal tree [5] modifies the traditional CART structure [25] to explicitly optimise for treatment effect heterogeneity and generates estimates at the leaves of the trees. Causal trees utilise a separate sample to detect the tree structure and another sample to estimate the treatment effects, this double-sample approach (also referred to as honest) helps to overcome the problem of over-fitting. Similar to the random forest for outcome prediction, each causal tree within the causal forest is built over a bootstrap sample from the training data and the forest averages over the tree generated treatment effects. In general, use of a forest over a large number of individual trees has been shown to more stable and produce more accurate results than an individual tree [21].

#### Sensitivity analysis for model evaluation in routine clinical data

We ran three sensitivity analysis to test the robustness of the CPRD validation: 1); as patients with a missing HbA1c outcome were excluded, we evaluated the potential influence of informative drop-out by repeating the validation incorporating inverse-probability of censoring weights (IPCW), with double robust adjustment for predictors and confounders in the outcome model. IPCW weights were derived from a logistic regression model with censoring status (Yes/No) as the outcome and incorporating all predictors and confounders described above including all patients with complete baseline data but allowing missing outcome data; 2); to evaluate the sensitivity of treatment effect outcomes to non-random treatment assignment we repeated the validation incorporating inverse-probability of treatment weights (IPTW), again derived from logistic regression incorporating all predictors and confounders and all patients with complete baseline data. IPCW were multiplied with IPTW and stabilised to define final model weights [26, 27]; 3) to assess whether differences in glycaemic outcome could reflect the fact DPP4i are most commonly prescribed at first intensification of glucose-lowering therapy (second-line, after initial metformin), while SGLT2i are most commonly third-line treatments, we repeated the primary model evaluation in the subset of patients initiating DPP4i or SGLT2i as second-line therapy only.

## Results

### Participant cohort

Baseline clinical characteristics of the trial cohort used for model development (*n* = 1,428) are reported in Table 2. 61 participants were excluded as they had no on-treatment HbA1c outcome available (sFlowchart 1). Mean achieved HbA1c at 26 weeks was 53.0 (SD 9.8) mmol/mol on SGLT2-i and 54.1 (SD 10.9) mmol/mol on DPP4-i.

**Table 2:** Baseline clinical characteristics by initiated drug class in CANTATA D and D2 randomised trials, and Clinical Practice Research Datalink (UK primary care data). Data are mean (SD) unless stated

	**Derivation set: CANTATA D and D2 trials**		**Validation set: Clinical Practice Research Datalink**	
	**SGLT2-inhibitor** **(*n* = 715)** **[Canagliflozin 300 mg]**	**DPP4-inhibitor** **(*n* = 713)** **[Sitagliptin 100 mg]**	**SGLT2-inhibitor** **(*n* = 11,682)** **[Any class]**	**DPP4-inhibitor** **(*n* = 7,059)** **[Any class]**
**Trial (n %)**				
CANTATA-D	355 (49.7)	356 (49.9)	NA	NA
CANTATA-D2	360 (50.3)	357 (50.1)	NA	NA
**Age (years)**	55.9 (9.4)	56.0 (9.4)	59.9 (9.1)	64.0 (10.8)
**Sex (n %)**				
Female	355 (49.7)	339 (47.5)	4,393 (37.6)	2,593 (36.7)
Male	360 (50.3)	374 (52.5)	7,289 (62.4)	4,466 (63.3)
**HbA1c (mmol/mol)**	63.9 (9.9)	63.9 (9.9)	76.8 (14.2)	72.4 (13.2)
**BMI (kg/m**^**2**^**)**	31.5 (6.6)	31.9 (6.5)	34.4 (6.6)	32.2 (6.4)
**eGFR (mL/min/1.3 m**^**2**^**)**	88.5 (18.2)	88.2 (19.5)	88.8 (14.4)	82.9 (17.2)
**HDL-c (mmol/L)**	1.2 (0.3)	1.2 (0.3)	1.1 (0.3)	1.2 (0.3)
**LDL-c (mmol/L)**	2.7 (0.9)	2.7 (0.9)	2.4 (1.0)	2.3 (0.9)
**Triglycerides (mmol/L)**	2.1 (1.4)	1.9 (1.2)	2.3 (1.4)	2.1 (1.3)
**ALT (IU/L)**	28.8 (18.5)	28.2 (14.7)	36.5 (44.2)	33.9 (56.9)
**Albumin (g/L)**	41.0 (3.3)	41.0 (3.3)	42.4 (4.0)	42.4 (3.9)
**Bilirubin (µmol/L)**	8.3 (4.0)	8.0 (0.9)	9.8 (5.0)	10.0 (5.1)
**Number of concurrent glucose-lowering drugs (n %)**				
0	0	0	187 (2.6)	665 (5.7)
1	355 (49.7)	356 (49.9)	2818 (39.9)	6947 (59.5)
2	360 (50.3)	357 (50.1)	3268 (46.3)	3914 (33.5)
3	0	0	786 (11.1)	156 (1.3)

### Model development

#### Penalized regression

In the development cohort the median average treatment effect was estimated as a 1.9 (IQR 0.5, 3.6) greater HbA1c reduction with SGLT2-i compared to DPP4-I (sFigure 1a). There was evidence of heterogeneity of treatment effect with a predicted greater HbA1c reduction with SGLT2-i versus DPP4-i for 1,216 (81.7%) of trial participants. Optimism-adjusted model performance statistics for predicting HbA1c outcome were: RMSE 8.1 (95%CI 7.6, 8.4) mmol/mol, R^2^ 0.30 (95%CI 0.26, 0.36), calibration slope 0.98 (95%CI 0.98, 1.00), calibration in the large 0.86 (95%CI -0.19. 0.95). Performance of the Lasso regression based model was similar (sTable 2), so only the primary penalised regression model was taken forward for external validation.

#### Causal forest

The median average treatment effect in the development cohort was a 1.6 (IQR 0.6, 2.5) greater HbA1c reduction with SGLT2-i therapy (sFigure 1b). Average treatment effects were consistent when varying the number of causal trees used to fit the model (sTable 3).There was evidence of heterogeneity in individual treatment effects (*p* = 0.005), although 1,408 (98.6%) of participants were predicted to have a greater benefit on SGLT2-i therapy.

### Model specification

#### Most influential predictors of differential treatment effect

**Fig. 1:**
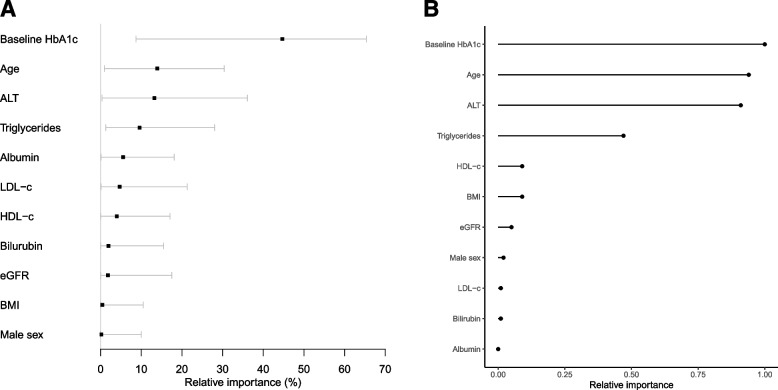
Relative feature importance for treatment selection with SGLT2-inhibitor and DPP4-inhibitor treatment, for all clinical features included in model development. **a** Penalized regression**.** Feature importance reflects the proportion of chi-squared explained by drug-by-covariate interaction terms for each clinical feature in multivariable analysis, as these represent differential treatment effects for the two therapies. Bars represent bootstrapped 95% confidence intervals. **b** Causal forest model. Adjusted importance (using *p*-values) represent the covariates selected most often by trees within the causal forest, after controlling for biased variable selection. Permutation-based tests generate *p*-values for each covariate, using an understanding that spurious splits in trees would continue to occur in the presence of a permuted outcome unless these splits also reflect the true underlying association. For the purpose of comparison, inverse *p*-values are presented as relative importance measures

Figure 1 reports the most influential predictors for differential treatment effect for the regression and causal forest approaches. Baseline HbA1c, age, ALT and triglycerides were the top 4 predictors identified by both approaches.

#### Model external validation: performance for treatment selection in routine clinical data

Utility for selecting treatment was evaluated in 18,741 patients initiating DPP4-i (*n* = 11,682), or SGLT2-i (*n* = 7,059) in CPRD (sFlowchart), after excluding 14,930 patients with missing outcome HbA1c and 8,136 patients with missing values for other predictors. Outcome HbA1c was observed at a median of 6 months (Interquartile range 4–8 months). Patients initiating each therapy differed in all clinical characteristics except sex and baseline albumin (Table 2). In particular, patients initiating DPP4-i were on average older than those initiating SGLT2-i (mean 64.0 versus 59.9 years), had a lower baseline HbA1c (mean 72.4 versus 76.8 mmol/mol), and had lower BMI (mean 32.2 versus 24.4 kg/m^2^) and eGFR (mean 82.9 versus 88.8 mL/min/1.3 m.^2^

**Fig. 2:**
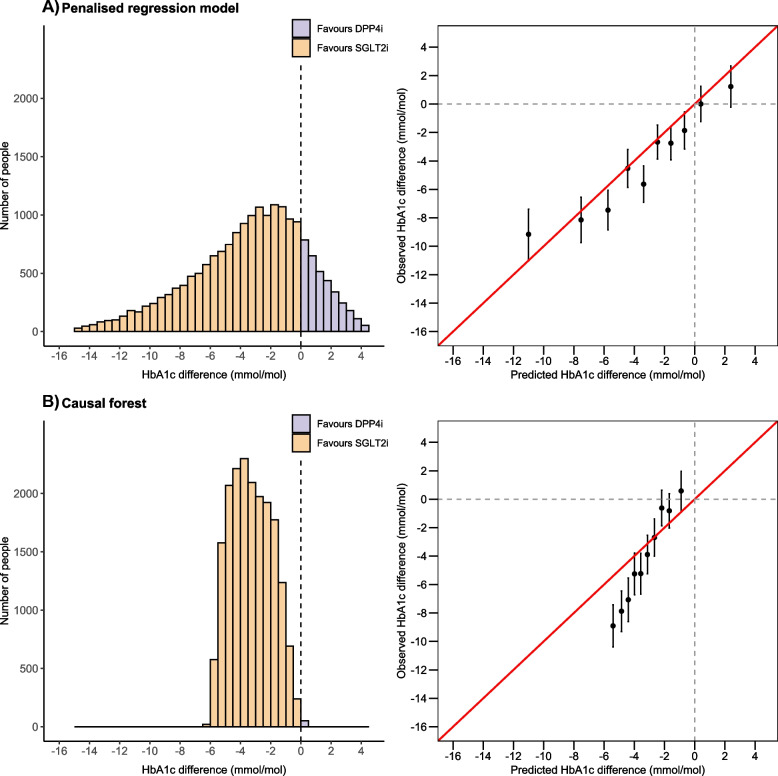
Final treatment selection model performance for **A** Penalized regression and **B** Causal forest in CPRD validation data. Left panels show the distribution of predicted individualized treatment effects. Negative values reflect a predicted benefit on SGLT2-inhibitor treatment, positive values reflect a predicted HbA1c benefit on DPP4-inhibitor treatment. Right panels show calibration between observed and predicted treatment effects, across strata defined by decile of predicted treatment effect. Estimates are adjusted for clinical features in the treatment selection model (see Methods: Predictors section), and potential confounders (see Methods: Confounders section) to improve precision and control for potential differences in covariate balance within strata

The distribution of model predicted treatment difference for the regression and causal forest approaches are shown in Fig. 2. The regression model predicted that 87% (*n* = 16,276) of patients would benefit on SGLT2-i and 13% (*n* = 2,465) on DPP4-i. In contrast, the causal forest model predicted that nearly all patients (99.7% [*n* = 18,689]) would benefit on a SGLT2-i.

From the regression model there was good calibration between observed and predicted estimates, across deciles of predicted treatment effect (Fig. 2, right panel). This included reliably identifying the smaller group of patients with a predicted treatment benefit on DPP4-i. Although the causal forest model did reliably identify patients with differences in observed treatment effect, the model did not show good calibration (Fig. 2). The causal forest predicted treatment effects were in a much narrower range than observed treatment effects, and the model did not identify a patient strata with an observed treatment benefit on DPP4-i. In strata defined by clinical cut-offs for predicted treatment benefit (Table 3), the regression model reliably identified 687 (3.7%) patients with a marked (≥ 10 mmol/mol) observed benefit on SGLT2-i. This group was not identified using the causal forest model. The regression model also identified a much larger group of patients with an observed benefit with SGLT2-i of 5–10 mmol/mol (*n* = 3,920 [20.9%]) compared to the causal forest model (n = 2,175 [11.6%]). Similarly, a group with a > 3 mmol/mol benefit on DPP4-i was identified with the regression model (*n* = 270 [1.4%]) but not the causal forest.

**Table 3:** External validation in CPRD: Observed treatment effects across strata defined by clinical cut-offs of predicted treatment benefit. Estimates are adjusted for clinical features in the treatment selection model (to improve precision and control for potential differences in covariate balance within subgroups)

	**Observed treatment difference (mmol/mol; negative favors SGLT2-i)**				
**Predicted HbA1c difference**	**N patients**	**Treatment difference (mean)**	**Lower 95% CI**	**Upper 95% CI**	***p*-value**
**Penalized regression model external validation**					
**Overall**	18,741	-4.5	-5.0	-4.1	< 0.001
**Strata**					
SGLT2-i benefit by any mmol/mol	15321	-5.2	-5.7	-4.7	< 0.001
SGLT2-i benefit by ≥ 10 mmol/mol	1173	-10.7	-13.0	-8.4	< 0.001
SGLT2-i benefit by 5–10 mmol/mol	4503	-7.6	-8.6	-6.6	< 0.001
SGLT2-i benefit by 3–5 mmol/mol	3517	-5.0	-5.9	-4.0	< 0.001
SGLT2-i benefit by 0–3 mmol/mol	6128	-2.3	-3.0	-1.6	< 0.001
DPP4-i benefit by any mmol/mol	3420	0.3	-0.7	1.3	0.506
DPP4-i benefit by 0–3 mmol/mol	2972	0.0	-1.0	1.1	0.937
DPP4-i benefit by ≥ 3 mmol/mol	448	2.9	-0.6	6.5	0.107
**Causal forest external validation**					
**Overall**	18741	-4.5	-4.9	-4.0	0.003
**Strata**					
SGLT2-i benefit by any mmol/mol	18689	-4.5	-5.0	-4.1	< 0.001
SGLT2-i benefit by ≥ 10 mmol/mol	0	NA	NA	NA	NA
SGLT2-i benefit by 5–10 mmol/mol	2175	-8.7	-10.0	-7.3	< 0.001
SGLT2-i benefit by 3–5 mmol/mol	8676	-6.0	-6.6	-5.3	< 0.001
SGLT2-i benefit by 0–3 mmol/mol	7838	-1.1	-1.7	-0.4	0.001
DPP4-i benefit by any mmol/mol	52	10.3	-8.6	29.3	0.269
DPP4-i benefit by 0–3 mmol/mol	52	10.3	-8.6	29.3	0.269
DPP4-i benefit by ≥ 3 mmol/mol	0	NA	NA	NA	NA

### Sensitivity analysis

Calibration of both models was similar when incorporating IPCW and IPTW into the CPRD model validation, and when restricting the CPRD cohort to patients initiating SGLT2-i or DPP4-I as second-line therapy (sFigures 2–4).

## Discussion

Our study provides a comparison of causal forest and regression approaches to detect and characterize treatment effect heterogeneity, as well as to operationalize it for treatment selection. Specifically, we observed that while both approaches detect treatment effect heterogeneity in glucose-lowering efficacy for SGLT2-i and DPP4-i, this translates into marked differences in predicted treatment benefit for individual patients. Through external validation using real-world (routinely collected) data, we establish the utility of both approaches for identifying strata with an observed benefit on one treatment over the other. We found a regression-based model performed substantially better than causal forest for identifying strata with a clinically important observed treatment benefit on SGLT2-i compared to DPP4-i. In contrast to causal forest, the regression model was also able to identify a smaller strata with a likely observed treatment benefit on DPP4-i.

From a methodological perspective, the analysis adds to the growing literature showing limited, if any, performance improvement for machine learning over regression in tasks utilizing structured clinical data [28–30], although our study provides important new evidence as previous evaluations have focused on performance for risk prediction rather than treatment effect heterogeneity. Whilst a recent simulation study found considerable advantages of causal forest over two-step regression (using two separate regression models to estimate treatment effects in each treatment arm) with non-linear and interactive covariates, and with high-dimensional covariates [7], no performance advantage was seen in our real-world evaluation. Indeed, in clinical trial data, with a limited set of candidate predictors reflecting parameters commonly measured in people with type 2 diabetes as part of their routine care, we found the causal forest algorithm outputted substantially more conservative estimates of treatment effect heterogeneity compared to penalized regression incorporating drug-predictor interactions and non-linearity for continuous predictors through the use of restricted cubic splines. In validation we then found that the greater treatment effect heterogeneity identified by the regression-based model was reproducible in real-world UK primary care data, whilst the causal forest based predictions were substantially miscalibrated. Although we demonstrate this with only a single outcome in a limited trial population, this reflects precisely the type of clinical dataset where machine learning methods for treatment effect heterogeneity are increasingly being deployed, for example in evaluation of risk of harm of intensive blood pressure management in the SPRINT trial [31], and evaluation of heterogeneity in mortality risk in people with diabetes in the ACCORD trial [32]. Given the lower performance of the causal forest algorithm in external validation, our study suggests that further research is urgently needed to understand the reasons underlying differences in outputs from treatment effect heterogeneity focused machine learning and regression based approaches in relatively low dimensional health datasets. In the meantime, we recommend that, when evaluating treatment effect heterogeneity, researchers do not rely on causal forest (or other similar machine learning) algorithms alone and compare outputs with standard regression. This is further supported by recent work suggesting subgroups defined by heterogenous treatment effects using causal trees may not be reproducible across randomized trials [33].

Moreover, in the specific context of type 2 diabetes management, our results support recent work showing that a ‘precision’ approach to treatment is possible by demonstrating clinically relevant heterogeneity of treatment response that can be predicted using simple patient characteristics and routine biomarker tests [13, 14]. Our findings raise the possibility of targeting specific treatment to patients most likely to have a greater HbA1c response, using characteristics that are already routinely measured. However, a limitation is that we evaluated only a single outcome, HbA1c. Treatment decisions are multi-factorial, and potential glycemic benefit should be considered alongside differences in side-effect profile, likely tolerability, and cardiovascular and renal benefit [12, 34]. Our study has the potential to inform future research to establish the potential utility of predicting individual-level treatment benefit for these outcomes.

Strengths of our study include the systematic comparison of both modelling approaches in the same datasets, and the use of individual-level trial data to develop treatment effect heterogeneity models, meaning randomization may allow a causal interpretation of individual-level treatment effects [35]. Whilst research to develop optimal methods for predicting treatment effect heterogeneity, and to evaluate their performance, has been called for in the recent PATH statement and is the subject of ongoing methodological development [2, 36], the evaluative framework applied in this study can be applied for any future study aiming to evaluate the value of using patient level features to inform a precision medicine approach to treatment in any disease with multiple treatment options [12].

A limitation of our study is that we only compared performance in a single, low dimensional setting with a continuous outcome; as shown in simulation-based analysis causal forest may outperform regression-based approaches with high dimensional or less structured data than those captured in clinical trial and routine clinical data [7]. The focus of our study was to systematically compare two different methods for modelling treatment effect heterogeneity using the same predictive features, and our candidate predictor set was informed by availability of features in both the RCT and observational data. It is therefore possible that we not to include all potentially influential predictors and research is ongoing to fully investigate robust predictors of treatment effect heterogeneity for these two drug classes [37]. A further limitation is that we only evaluated a single machine learning approach. Causal forest was chosen as it is widely used with easy to use software available. Our focus was not a comprehensive review of all closely related methods, and so we cannot comment on the performance of other treatment effect heterogeneity focused algorithms, in particular recently developed Bayesian approaches [38–40] Finally, as our validation dataset was observational, despite extensive sensitivity analysis we cannot rule out unmeasured confounding as a potential explanation for our findings [41].

## Conclusions

The causal forest machine learning algorithm is outperformed by standard regression when identifying patients with type 2 diabetes with a treatment benefit if receiving one blood glucose-lowering drug over another. Given the rapidly growing interest in precision medicine, further research is urgently needed to understand the settings in which different classical and data-driven modelling approaches can be effectively deployed to reliably detect and quantify treatment effect heterogeneity.

## Supplementary Information

**Additional file 1: sFlowchart.** A) CANTATA D and D2 trials. B) CPRD patient flow and inclusion criteria. **sTable 1.** Additional baseline clinical characteristics by initiated drug class in CPRD. **sFigure 1.** Distribution of the predicted individualized treatment effect of SGLT2-inhibitor treatment compared to DPP4-inhibitor treatment in the RCT derivation data. a) Penalized regression. **sTable 2.** Comparison of model performance for the penalised ridge regression model with lasso regression. **sTable 3.** Sensitivity analysis testing variation in predicted treatment effects varying the number of trees in the causal forest algorithm. **sFigure 2.** Treatment selection model performance for A) Penalized regression and B) Causal forest in CPRD validation data using double robust estimation incorporating inverse probability of treatment weighting and adjustment for all predictors and confounders in the outcome model. **sFigure 3.** Treatment selection model performance for A) Penalized regression and B) Causal forest in CPRD validation data using double robust estimation incorporating inverse probability of treatment weighting, inverse probability of censoring weighting, and adjustment for all predictors and confounders in the outcome model. **sFigure 4.** Treatment selection model performance for A) Penalized regression and B) Causal forest in CPRD validation data, in patients initiating second-line therapy only. **TRIPOD Checklist.**

## Data Availability

The routine clinical data analysed during the current study are available in the CPRD repository (CPRD; https://cprd.com/research-applications), but restrictions apply to the availability of these data, which were used under license for the current study, and so are not publicly available. For re-using these data, an application must be made directly to CPRD. The clinical trial data analysed during the current study are available in the Yale University Open Data Access Project repository (YODA; https://yoda.yale.edu/how-request-data); these data are not publicly available and to re-use these data an application must be made directly to YODA.

## Abbreviations

ACCORD: The Action to Control Cardiovascular Risk in Diabetes trial
ALT: Alanine Aminotransferase
BMI: Body mass index
CATE: Conditional average treatment effect
CPRD: Clinical Practice Research Datalink
DPP4: Dipeptidyl peptidase 4
eGFR: Estimated glomerular filtration rate
HbA1c: Glycated Haemoglobin A1c
HDL-c: High-density lipoprotein cholesterol
LDL-c: Low-density lipoprotein cholesterol
RCT: Randomized controlled trial
RMSE: Root mean square error
SGLT2: Sodium-glucose co-transporter-2
SPRINT: The Systolic Blood Pressure Intervention Trial
