# Supplementary material for: Comparison of causal forest and regression-based approaches to evaluate treatment effect heterogeneity: an application for type 2 diabetes precision medicine
Source: BMC Med Inform Decis Mak. 2023 Jun 16;23:110. doi: 10.1186/s12911-023-02207-2 (PMC10276367; doi:10.1186/s12911-023-02207-2)
Supplement: Supplementary file 1 — Additional file 1: sFlowchart. A) CANTATA D and D2 trials. B) CPRD patient flow and inclusion criteria. sTable 1. Additional baseline clinical characteristics by initiated drug class in CPRD. sFigure 1. Distribution of the predicted individualized treatment effect of SGLT2-inhibitor treatment compared to DPP4-inhibitor treatment in the RCT derivation data. a) Penalized regression. sTable 2. Comparison of model performance for the penalised ridge regression model with lasso regression. sTable 3. Sensitivity analysis testing variation in predicted treatment effects varying the number of trees in the causal forest algorithm. sFigure 2. Treatment selection model performance for A) Penalized regression and B) Causal forest in CPRD validation data using double robust estimation incorporating inverse probability of treatment weighting and adjustment for all predictors and confounders in the outcome model. sFigure 3. Treatment selection model performance for A) Penalized regression and B) Causal forest in CPRD validation data using double robust estimation incorporating inverse probability of treatment weighting, inverse probability of censoring weighting, and adjustment for all predictors and confounders in the outcome model. sFigure 4. Treatment selection model performance for A) Penalized regression and B) Causal forest in CPRD validation data, in patients initiating second-line therapy only. TRIPOD Checklist. [file 12911_2023_2207_MOESM1_ESM.pdf]

Supplementary material for:

Comparison of causal forest and regression-based approaches to evaluate treatment effect heterogeneity: An application for type 2 diabetes precision medicine

Authors: Ashwini Venkatasubramaniam, Bilal A. Mateen, Beverley M Shields, Andrew T Hattersley, Angus G Jones, Sebastian J. Vollmer, John M. Dennis

sFlowchart

A) CANTATA D and D2 trials (development cohort)

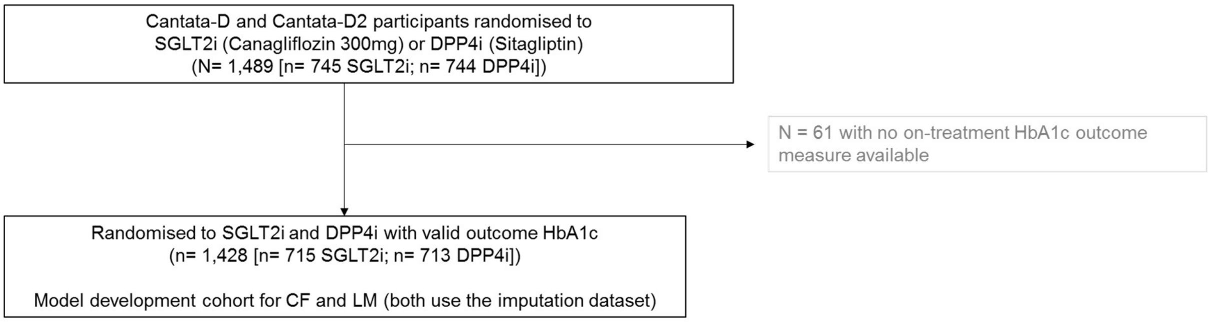

B) CPRD patient flow and inclusion criteria (validation cohort)

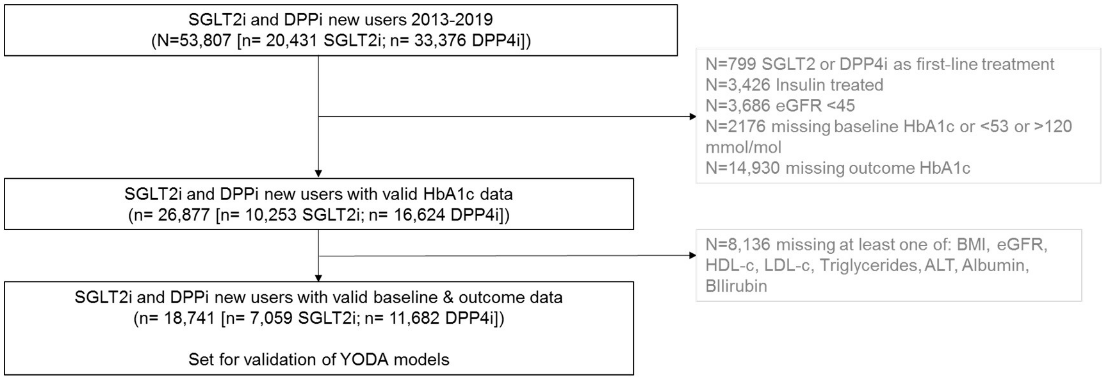

sTable 1: Additional baseline clinical characteristics by initiated drug class in CPRD. Data are mean (SD) unless stated.

	Validation set: Clinical Practice Research Datalink	
	**SGLT2-inhibitor (n=11,682) [Any class]**	**DPP4-inhibitor (n=7,059) [Any class]**
**Number of previous glucose-lowering drug classes**		
1	1473 (20.9)	5305 (45.4)
2	2235 (31.7)	4818 (41.2)
3	2078 (29.4)	1309 (11.2)
4+	1273 (18.0)	250 (2.1)
**Diabetes duration (years)**	8.5 (5.1)	8.0 (5.3)
**Non-adherent on first-line therapy (n %)**	993 (14.1)	1499 (12.8)
**Ethnicity (n %)***		
White	3127 (88.6)	5447 (88.2)
Asian	261 (7.4)	466 (7.5)
Black	73 (2.1)	145 (2.3)
Mixed or Other	67 (1.9)	116 (1.9)
**Smoking status**		
Active smoker	989 (14.0)	1509 (12.9)
Ex-smoker	2597 (36.8)	4421 (37.8)
Non-smoker	3473 (49.2)	5752 (49.2)
**Cardiorenal comorbidities**		
Hypertension	3947 (55.9)	6768 (57.9)
Cardiovascular disease**	1143 (16.2)	2352 (20.1)
Heart failure	139 (2.0)	362 (3.1)
Chronic kidney disease	203 (2.9)	1399 (12.0)
**Microvascular complications**		
Neuropathy	1026 (14.5)	1954 (16.7)
Nephropathy	135 (1.9)	252 (2.2)
Retinopathy	2048 (29.0)	3200 (27.4)

*Ethnicity was not available for 3,531 patients initiating SGLT2-inhibitors and 5,508 patients initiating DPP4-inhibitors. Ethnicity was adjusted for in risk models using the missing category method.

**Composite of history of: myocardial infarction, stroke, ischaemic heart disease, peripheral artery disease, revascularisation.

sFigure 1: Distribution of the predicted individualized treatment effect of SGLT2-inhibitor treatment compared to DPP4-inhibitor treatment in the RCT derivation data. a) Penalized regression. SGLT2-i was the predicted optimal therapy for 1,216 (85.1%) participants, DPP4-i for 212 (14.8%) participants. **b) Causal forest.** SGLT2-i was the predicted optimal therapy for 1,414 (99.0%) participants, DPP4-i for 14 (1.0%) of participants

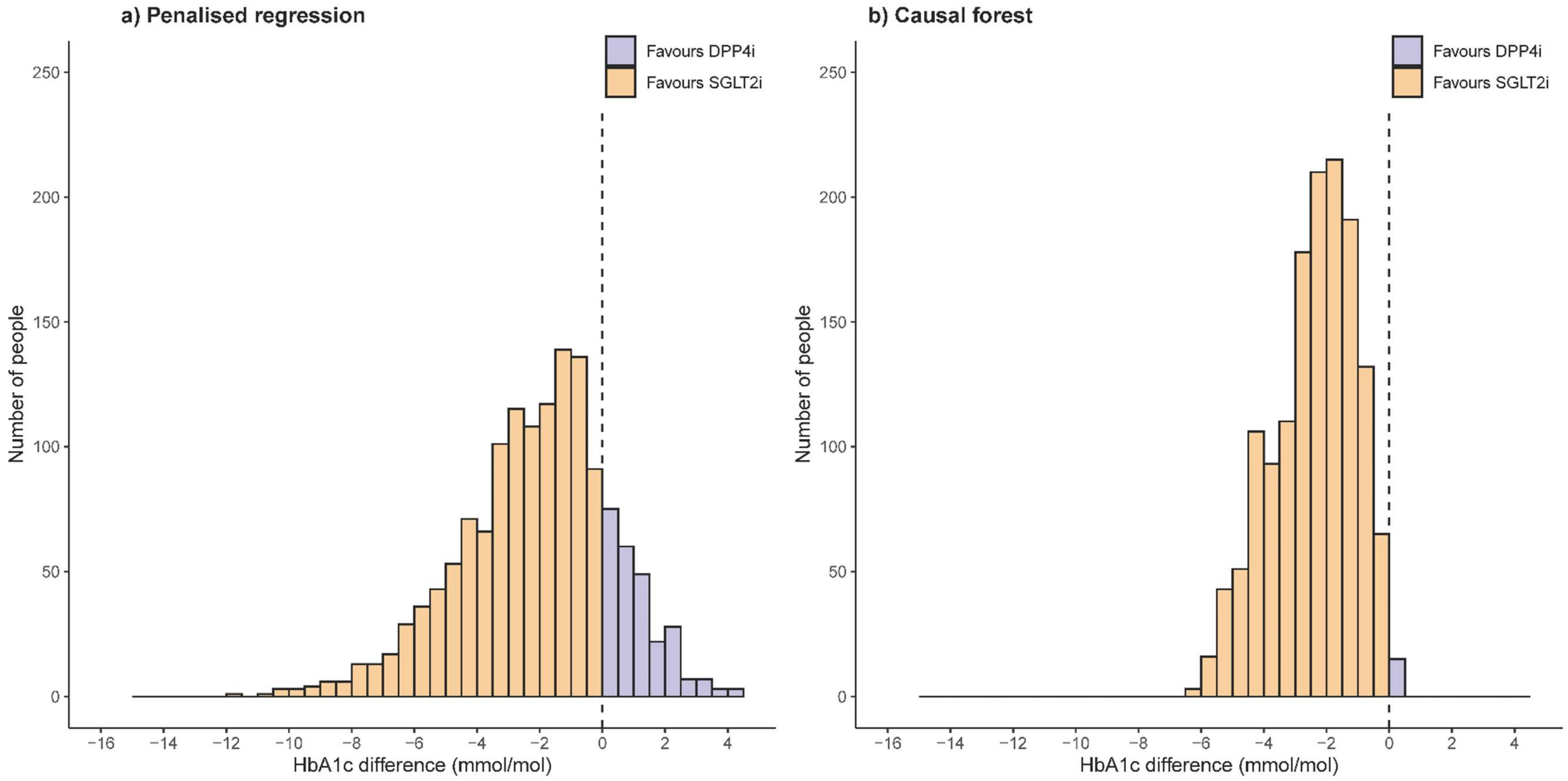

sTable 2: Comparison of model performance for the penalised ridge regression model with lasso regression

Performance measure	Penalised ridge regression*	Lasso regression (1se)**	Lasso regression (min)***
RMSE (mmol/mol)	8.0 (95%CI 7.6, 8.4)	8.2 (95%CI 7.9, 8.7)	8.0 (95%CI 7.6, 8.3)
R^2	0.31 (95%CI 0.26, 0.36)	0.28 (95%CI 0.24, 0.32)	0.31 (95%CI 0.27, 0.36)
Calibration slope	0.99 (95%CI 0.98, 1.00)	1.33 (95%CI 1.20, 1.46)	1.05 (95%CI 0.95, 1.15)

* Optimism adjusted performance with 1000 bootstraps
** Largest value of lambda such that error is within 1 standard error of cross-validated errors, in 1000-fold cross-validation
*** At minimum mean cross-validated error, in 1000-fold cross-validation

sTable 3: Sensitivity analysis testing variation in predicted treatment effects varying the number of trees in the causal forest algorithm.

Number of trees used in causal forest algorithm	Minimum average treatment effect* (mmol/mol)	Median (IQR) average treatment effect (mmol/mol)*	Maximum average treatment effect* (mmol/mol)
50	-5.9	-1.7 (-2.6, -0.8)	1.4
100	-5.4	-1.5 (-2.4, -0.7)	2.0
200	-4.7	-1.4 (-2.3, -0.6)	1.4
500	-5.1	-1.5 (-2.5, -0.7)	1.2
1000	-4.8	-1.5 (-2.5, -0.8)	1.1
2500	-4.7	-1.6 (-2.5, -0.8)	1.2
5000 (primary model)	-4.8	-1.6 (-2.5, -0.8)	1.1
7500	-4.8	-1.5 (-2.5, -0.8)	1.1
10000	-4.8	-1.6 (-2.5, -0.8)	1.1

sFigure 2: Treatment selection model performance for A) Penalized regression and B) Causal forest in CPRD validation data using double robust estimation incorporating inverse probability of treatment weighting and adjustment for all predictors and confounders in the outcome model (n=18,741). Left panels show the distribution of predicted individualized treatment effects. Negative values reflect a predicted benefit on SGLT2-inhibitor treatment, positive values reflect a predicted HbA1c benefit on DPP4-inhibitor treatment. Right panels show calibration between observed and predicted treatment effects, across strata defined by decile of predicted treatment effect.

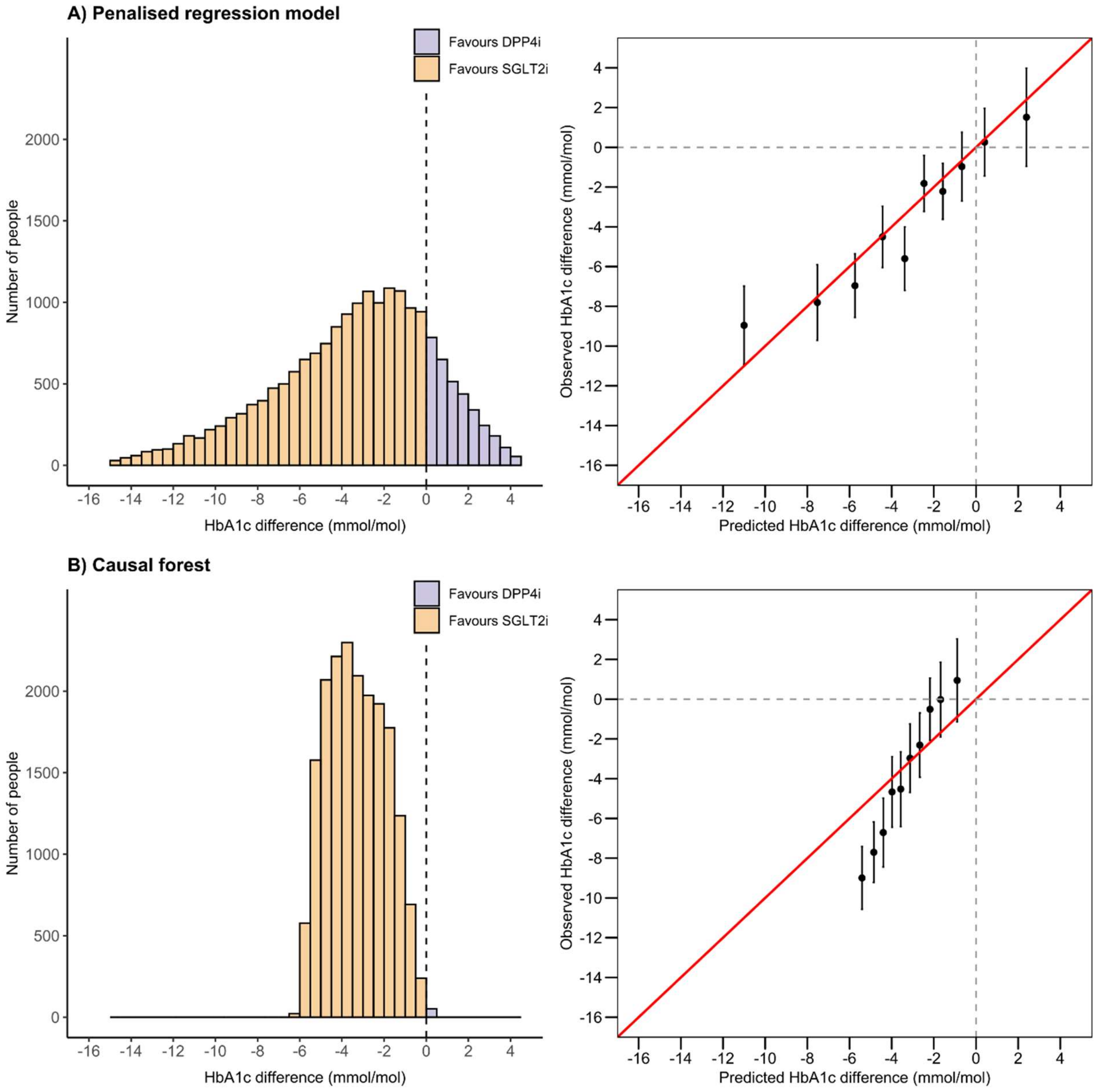

sFigure 3: Treatment selection model performance for A) Penalized regression and B) Causal forest in CPRD validation data using double robust estimation incorporating inverse probability of treatment weighting, inverse probability of censoring weighting, and adjustment for all predictors and confounders in the outcome model (n=18,741). Left panels show the distribution of predicted individualized treatment effects. Negative values reflect a predicted benefit on SGLT2-inhibitor treatment, positive values reflect a predicted HbA1c benefit on DPP4-inhibitor treatment. Right panels show calibration between observed and predicted treatment effects, across strata defined by decile of predicted treatment effect.

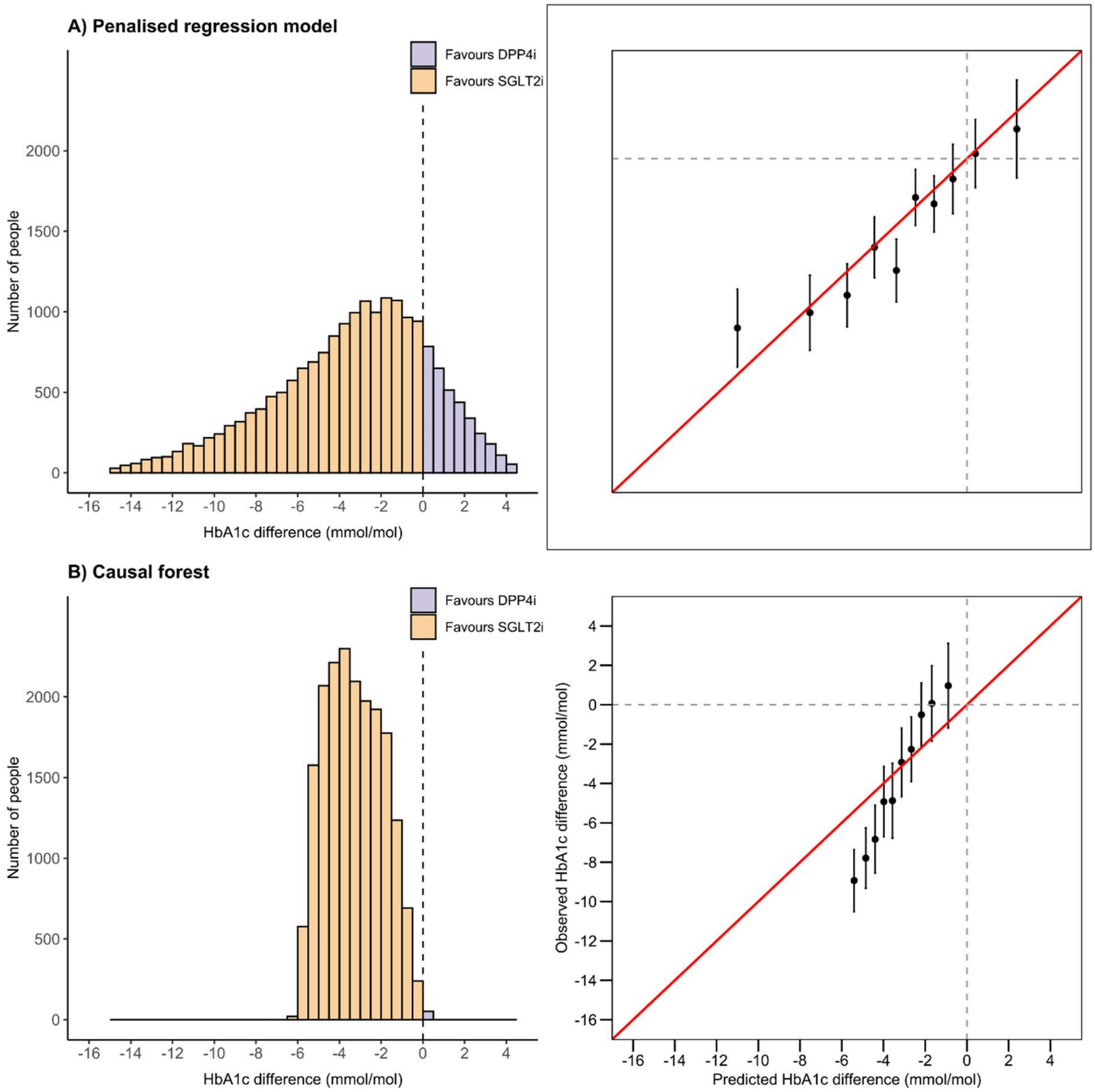

sFigure 4: Treatment selection model performance for A) Penalized regression and B) Causal forest in CPRD validation data, in patients initiating second-line therapy only (n=1,473 patients initiating SGLT2-inhibitors and n=5,305 patients initiating DPP4-inhibitors). Left panels show the distribution of predicted individualized treatment effects. Negative values reflect a predicted benefit on SGLT2-inhibitor treatment, positive values reflect a predicted HbA1c benefit on DPP4-inhibitor treatment. Right panels show calibration between observed and predicted treatment effects, across strata defined by decile of predicted treatment effect. Estimates are adjusted for clinical features in the treatment selection model, and confounders (see Methods) to improve precision and control for potential differences in covariate balance within strata.

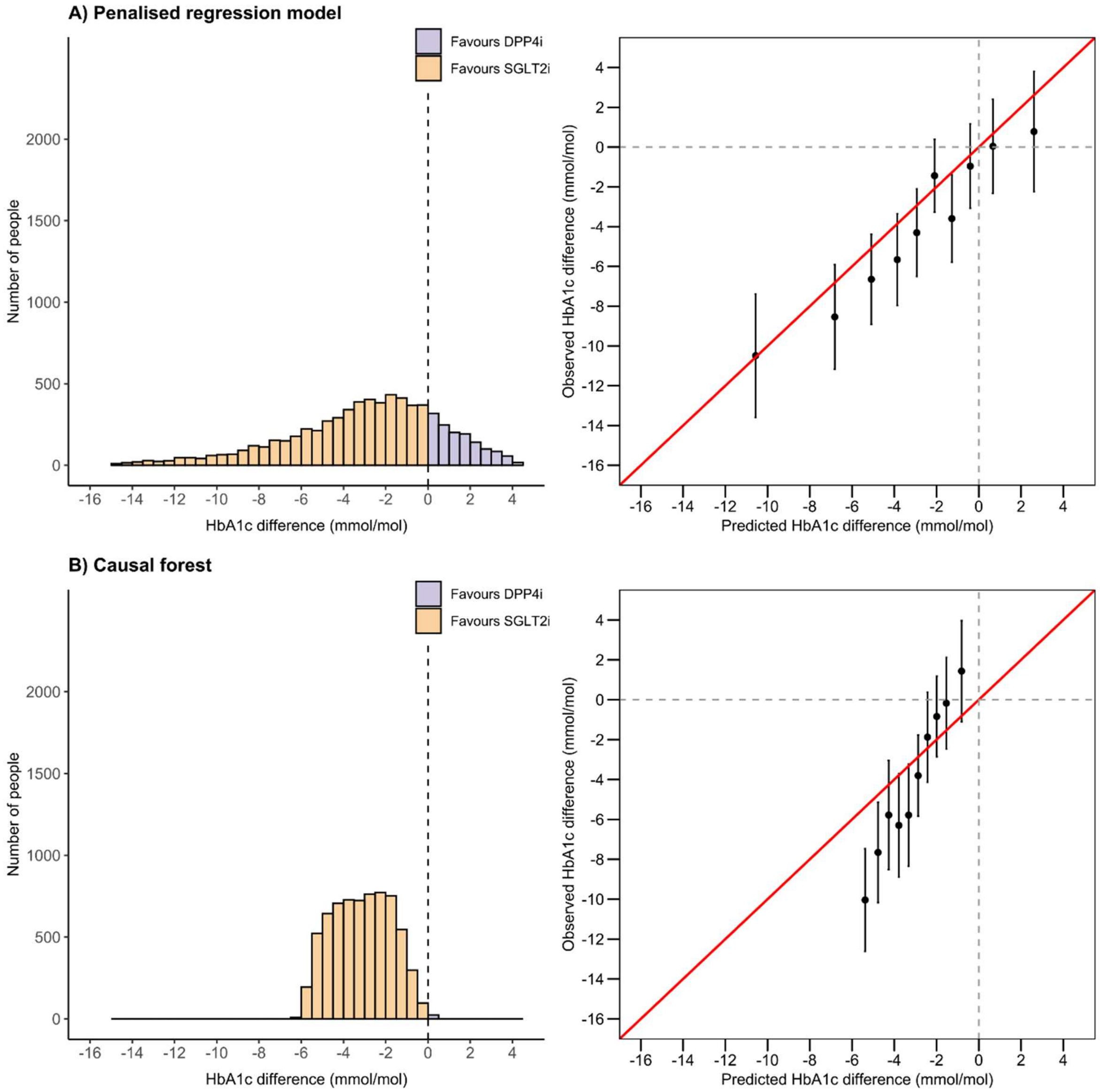

TRIPOD Checklist: Prediction Model Development

Section/Topic	Item	Checklist Item	Page
**Title and abstract**			
Title	1	Identify the study as developing and/or validating a multivariable prediction model, the target population, and the outcome to be predicted.	1
Abstract	2	Provide a summary of objectives, study design, setting, participants, sample size, predictors, outcome, statistical analysis, results, and conclusions.	3
**Introduction**			Section, Paragraph
Background and objectives	3a	Explain the medical context (including whether diagnostic or prognostic) and rationale for developing or validating the multivariable prediction model, including references to existing models.	Introduction, para 1 and 2
	3b	Specify the objectives, including whether the study describes the development or validation of the model or both.	Introduction, para 3
**Methods**			
Source of data	4a	Describe the study design or source of data (e.g., randomized trial, cohort, or registry data), separately for the development and validation data sets, if applicable.	Methods, paras 1, 2 and 3
	4b	Specify the key study dates, including start of accrual; end of accrual; and, if applicable, end of follow-up.	Methods, paras 2 and 3
Participants	5a	Specify key elements of the study setting (e.g., primary care, secondary care, general population) including number and location of centres.	Methods, paras 2 and 3
	5b	Describe eligibility criteria for participants.	Methods 2 & 3
	5c	Give details of treatments received, if relevant.	Methods 1
Outcome	6a	Clearly define the outcome that is predicted by the prediction model, including how and when assessed.	Methods 4
	6b	Report any actions to blind assessment of the outcome to be predicted.	Methods 2
Predictors	7a	Clearly define all predictors used in developing or validating the multivariable prediction model, including how and when they were measured.	Methods 5 and 6
	7b	Report any actions to blind assessment of predictors for the outcome and other predictors.	Methods 2
Sample size	8	Explain how the study size was arrived at.	Methods 2 & 3
Missing data	9	Describe how missing data were handled (e.g., complete-case analysis, single imputation, multiple imputation) with details of any imputation method.	Methods 7
Statistical analysis methods	10a	Describe how predictors were handled in the analyses.	Methods 9 & 10
	10b	Specify type of model, all model-building procedures (including any predictor selection), and method for internal validation.	Methods 9 & 10
	10d	Specify all measures used to assess model performance and, if relevant, to compare multiple models.	Methods 11 & 12
Risk groups	11	Provide details on how risk groups were created, if done.	Methods: Model evaluation
**Results**			
Participants	13a	Describe the flow of participants through the study, including the number of participants with and without the outcome and, if applicable, a summary of the follow-up time. A diagram may be helpful.	sFlowchart 1
	13b	Describe the characteristics of the participants (basic demographics, clinical features, available predictors), including the number of participants with missing data for predictors and outcome.	Table 1 Results 1
Model development	14a	Specify the number of participants and outcome events in each analysis.	Results 2 & 3
	14b	If done, report the unadjusted association between each candidate predictor and outcome.	NA
Model specification	15a	Present the full prediction model to allow predictions for individuals (i.e., all regression coefficients, and model intercept or baseline survival at a given time point).	Results 4, 5, 6 and 7 Figure 1 Table 2
	15b	Explain how to the use the prediction model.	Results 5, 6 and 7
Model performance	16	Report performance measures (with CIs) for the prediction model.	Results 7 Figure 2
**Discussion**			
Limitations	18	Discuss any limitations of the study (such as nonrepresentative sample, few events per predictor, missing data).	Discussion, 3 and 5
Interpretation	19b	Give an overall interpretation of the results, considering objectives, limitations, and results from similar studies, and other relevant evidence.	Discussion 1, 2, and 3
Implications	20	Discuss the potential clinical use of the model and implications for future research.	Discussion, 2 & 4
**Other information**			
Supplementary information	21	Provide information about the availability of supplementary resources, such as study protocol, Web calculator, and data sets.	Acknowledgements
Funding	22	Give the source of funding and the role of the funders for the present study.	Acknowledgements

We recommend using the TRIPOD Checklist in conjunction with the TRIPOD Explanation and Elaboration document.
